# The Conflict between Cheetahs and Humans on Namibian Farmland Elucidated by Stable Isotope Diet Analysis

**DOI:** 10.1371/journal.pone.0101917

**Published:** 2014-08-27

**Authors:** Christian C. Voigt, Susanne Thalwitzer, Jörg Melzheimer, Anne-Sophie Blanc, Mark Jago, Bettina Wachter

**Affiliations:** 1 Department of Evolutionary Ecology, Leibniz Institute for Zoo and Wildlife Research, Berlin, Germany; 2 Institute of Zoology, University of Neuchâtel, Neuchâtel, Switzerland; 3 The AfriCat Foundation, Farm Okonjima, Otjiwarongo, Otjozondjupa, Namibia; University of Western Ontario, Canada

## Abstract

Large areas of Namibia are covered by farmland, which is also used by game and predator species. Because it can cause conflicts with farmers when predators, such as cheetahs (*Acinonyx jubatus*), hunt livestock, we assessed whether livestock constitutes a significant part of the cheetah diet by analysing the stable isotope composition of blood and tissue samples of cheetahs and their potential prey species. According to isotopic similarities, we defined three isotopic categories of potential prey: members of a C4 food web with high δ^15^N values (gemsbok, cattle, springhare and guinea fowl) and those with low δ^15^N values (hartebeest, warthog), and members of a C3 food web, namely browsers (eland, kudu, springbok, steenbok and scrub hare). We quantified the trophic discrimination of heavy isotopes in cheetah muscle in 9 captive individuals and measured an enrichment for ^15^N (3.2‰) but not for ^13^C in relation to food. We captured 53 free-ranging cheetahs of which 23 were members of groups. Cheetahs of the same group were isotopically distinct from members of other groups, indicating that group members shared their prey. Solitary males (*n* = 21) and males in a bachelor groups (*n* = 11) fed mostly on hartebeest and warthogs, followed by browsers in case of solitary males, and by grazers with high δ^15^N values in case of bachelor groups. Female cheetahs (*n* = 9) predominantly fed on browsers and used also hartebeest and warthogs. Mixing models suggested that the isotopic prey category that included cattle was only important, if at all, for males living in bachelor groups. Stable isotope analysis of fur, muscle, red blood cells and blood plasma in 9 free-ranging cheetahs identified most individuals as isotopic specialists, focussing on isotopically distinct prey categories as their food.

## Introduction

Over past years, evidence has accumulated that local extirpation of top predators may have severe and unforeseen consequences for the structure and functioning of whole ecosystems [1]–[3]. Therefore, improved management strategies require a detailed knowledge about the trophic linkages between predators and their prey, particularly because many top predators face an uncertain future in anthropogenic shaped ecosystems [4], [5]. Indeed, most large predator species have been eradicated in areas with anthropogenic influences such as African farmland which is used for intensive livestock and/or game production [6]. Even though Namibia hosts the largest population of cheetahs (*Acinonyx jubatus*) worldwide [7], this population is threatened by farmers who perceive cheetahs as a threat to their animals [6]. Some farmers even preventively kill cheetahs when facing losses of livestock or game species important for trophy hunting [6], [8]. From a conservation point of view, it is therefore crucial to understand the diet of Namibian cheetahs and to assess whether livestock or large game constitute a significant part of their diet.

Cheetahs hunt all their prey by themselves and do not kleptoparasitize from other predators or scavenge from carcasses [9], [10]. Thus, it is valid to assume that the species identified indirectly as a prey animal was killed by the cheetah, even in the absence of direct observations. Previous dietary studies based on visual inspection of undigested matter in fecal samples indicated that cheetahs hunt mostly small to medium-sized mammals, and occasionally also birds [11], [12]. Also, these studies suggested that Namibian cheetahs do not prey largely on livestock species such as cattle, goat or sheep. Even though this indirect approach is valuable and was refined over past years [13], it provides only a snapshot of the dietary composition of cheetahs and not a quantitative approach that integrates over a longer period. Since the measurement of stable isotope ratios promises to meet the criteria of an integrative and quantitative approach [14], [15], we decided to investigate the diet of cheetahs by looking at stable carbon and nitrogen isotope ratios of their tissues. A stable isotope approach seems particularly promising because it enables investigators to assess the degree of specialization in consumers using the isotopic similarity of tissues with different isotopic retention time [16] or that of whiskers or tail hair increments [17].

Plants of southern Africa are known to differ in stable carbon isotope ratios because of variations in their CO_2_ photosynthetic pathway [18], [19]. Thus, herbivores feeding on grasses have a higher stable carbon isotope ratio in their tissue than herbivores feeding on browse [20]–[27]. Based on previous stable isotope studies in sub-Saharan African ecosystems, we hypothesized that potential prey species of cheetahs can be assigned to food webs that carry a carbon isotope signature of either C3 plants or C4 plants. Accordingly, we predicted that browsing herbivores in our study such as eland (*Taurotragus oryx*), kudu (*Tragelaphus strepsiceros*), springbok (*Antidorcas marsupialis*), steenbok (*Raphicerus campestris*) and scub hare (*Lepus saxatilis*) will have lower stable carbon isotope ratios than grazing herbivores such as gemsbok (*Oryx gazella*), hartebeest (*Alcelaphus buselaphus*), cattle (*Bos taurica*), warthog (*Phacochoerus africanus*) and springhare (*Pedetes capensis*). We also included guinea fowls (*Numida meleagris*) in our study, because previous studies suggested that cheetahs feed occasionally on these birds [6]. Given the preference of guinea fowl on grass seeds and grass-processing insects such as termites, we expected that guinea fowl tissue should be similar in stable isotope composition to those of grazing ungulates. We expected potential prey species to group in isotopic clusters according to their isotopic similarity.

With respect to the diet of cheetahs, we formulated three hypotheses. Firstly, we expected that members of the same social unit, such as bachelor groups, are more similar in isotopic composition than those of other social units, because members of the same group are likely to share prey. Secondly, we assumed that solitary males, male bachelor groups and females feed on different prey sizes or age classes because males are heavier than females and therefore are likely to subdue larger prey sizes and also calves of large prey species defended by their mothers [9], [10], [28]. Therefore, we expected that females mainly hunt prey species of those isotopic clusters that contain mainly small and medium-sized adult prey species such as springbok or steenbok. Thirdly, we used a replicate stable isotope approach to study the degree of dietary specialization in individual cheetahs. Cheetah individuals might be isotopic specialists because of a patchy distribution of prey species or because of sex- or age-specific hunting behaviors. For this part of our study, we collected four tissue or body products that differ in isotopic retention time. This approach takes advantage of the fact that tissues differ in the turnover of stable isotopes according to their metabolic or growth rate and therefore integrate stable isotopes over varying periods. We analyzed stable isotope ratios in fur, muscle, red blood cells and blood plasma (sorted according to decreasing isotopic retention). We defined cheetahs as isotopic specialists when they focused on a specific isotopic prey category and when their tissues were similar in isotopic composition [29], [30]. If cheetahs are isotopic specialists, we predict that within-individual variance of stable isotope ratios in various tissue samples is low and that the majority of isotopic variance is explained by between-individual variation [31].

## Materials and Methods

### Field work and ethics statement

Field work was conducted on cattle and game farmland in central Namibia between June 2002 and June 2007, and tissue samples for the isotopic discrimination in cheetah tissue were collected between September 2006 and December 2006 at the AfriCat Foundation, a non-profit carnivore conservation facility housing a limited number of captive cheetahs in central Namibia. Our study was approved by the Ministry of Environment and Tourism (MET) in Windhoek, Namibia (permit numbers 525/2002, 700/2003, 764/2004, 939/2005, 1089/2006, 1194/2007) and the Animal Care and Ethics Committee of the Leibniz Institute for Zoo and Wildlife Research in Berlin.

### Isotopic analysis of potential prey

#### Collection of tissue material from potential prey species

We obtained muscle tissue of game species from fresh kills of predators, from trophy hunters who hunted legally on farmland and from farmers who killed animals for their own consumption. Cattle samples were collected from individuals at the slaughterhouse of the Meat Cooperation of Namibia in Windhoek. We collected samples from adult individuals of nine prey species which comprise a large proportion of prey species of cheetahs in the study site [11], [12]. We did not collect samples from nursing offspring or juveniles of these prey species because stable isotope ratios are known to be similar between mother and offspring [32]. Prey species were gemsbok (*n* = 30), hartebeest (*n* = 15), cattle (*n* = 28), warthog (*n* = 14), guinea fowl (*n* = 4), springhare (n = 10), eland (*n* = 2), kudu (*n* = 10), springbok (*n* = 7), steenbok (*n* = 2) and scrub hare (*n = 3*). They were selected according to their relevance in the human-wildlife conflict concerning livestock (cattle) and trophy species (kudu, eland, gemsbok), based on the literature and on availability. The latter aspect precluded the inclusion of common duiker (*Silvicapra grimmia*), which is also known to be hunted by Namibian cheetahs [11], [12]. Small livestock such as goat and sheep might also be of relevance in the human-wildlife conflict, however, in our study area the most important livestock species is cattle, thus we concentrated on this species. All samples were air-dried in the field, stored in cryo-vials and shipped to the stable isotope laboratory of the Department of Geology and Mineralogy of the University of Erlangen-Nuremberg.

#### Stable isotope analysis

All samples were dried in a drying oven over 24 hours at 50°C. We put 0.5 mg (±0.1 mg) from each sample in tin capsules (IVA Analysetechnik e.K. Meerbusch, Germany) and placed the capsules in an autosampler. Samples were combusted and analyzed using an elemental analyzer (CE 1110 EA; Thermo Finnigan, Bremen, Germany) coupled to a Delta Plus isotope ratio mass spectrometer (Thermo Finnigan) in continuous flow. Atmospheric nitrogen was used as the standard for stable nitrogen isotopes and Vienna-PDB for stable carbon isotopes. The methodological approach in analyzing stable carbon and nitrogen isotope ratios has been described in detail in [33]. All stable isotope ratios are expressed in the delta notation as parts per mille deviations of the sample isotope ratios from the ratios of respective standards. Precision of measurements was always better than 0.4‰ (one standard deviation; SD).

#### Distinction of isotopic prey categories

In order to assign prey species to isotopically similar prey categories, we used a combination of statistical analysis and evaluation of pair-wise isotopic differences. The underlying reason for this is that the isotopic composition of two prey species might proof statistically significantly different, yet this outcome may be generated by a relatively large sample size and low variance. In other cases, isotopic differences between two species might not be detectable by statistical means because of large variance and low sample size. Therefore, we used our statistical approach as a guide for assigning potential prey species to isotopic categories.

For statistical comparisons, we square root transformed unsigned delta values to meet normal distribution. We then produced a Bray–Curtis similarity matrix of prey species, followed by an ANOSIM to identify potential differences in isotopic values between prey categories [34]. Then, we performed a SIMPER analysis, which quantifies the percentage of isotopic dissimilarity between pairs of species and depicts the sample category which was most relevant in explaining the dissimilarity. For these analyses, we used Primer 6 (Version 6-1-15; Primer-E Ltd.) and assumed an alpha value of 0.05. All parameters are presented as means ± one SD throughout the manuscript.

### Diet of cheetahs on Namibian farmland

#### Isotopic discrimination in cheetah tissues

To quantify the isotopic discrimination between cheetah and donkey tissue, nine cheetahs were fed at AfriCat for at least two months with only meat of donkey (*Equus asinus asinus*). We obtained muscle tissue from seven donkeys for comparative purposes. From each cheetah we collected a small sample of muscle tissue from the hind leg by mechanically restraining the animals and using a biopsy needle. Samples were sun-dried, stored in cryo-vials and shipped to the stable isotope laboratory at the University of Erlangen-Nuremberg, where they were analyzed as outlined above. We tested for isotopic differences in muscle tissue of cheetahs and donkeys with a Mann-Whitney-U-test.

#### Sample collection in free-ranging cheetahs and stable isotope analysis

We collected blood samples of 53 free-ranging cheetahs (44 males, 9 females) to determine their diet composition using stable isotopes. Cheetahs were captured at cheetah marking trees, immobilized, sampled and collared as described in [35], [36]. Venous blood was collected into EDTA blood tubes (BD Vacutainer Systems, Plymouth, United Kingdom), except in one case when we had access to a fresh carcass that was shot as a so-called problem animal and we collected blood from the heart ventricle. Blood samples were kept at 4°C during transport to the field station and were centrifuged at 5,000 rpm for 15 min. Red blood cells (RBC) were stored at −196°C in a liquid nitrogen container and then transported to the stable isotope laboratory at the Leibniz-Institute for Zoo and Wildlife Research (IZW). There, RBC were dried in a drying oven until constant mass and analysed for stable isotope ratios. Analytical procedures were similar to the ones described above except that samples were combusted and analyzed using an elemental analyzer (Flash EA; Thermo Finnigan, Bremen, Germany) coupled to a Delta V advantage isotope ratio mass spectrometer (Thermo Finnigan) in a continuous flow. Stable isotope ratios were expressed in the delta-notation in relation to international standards.

We categorized cheetahs as either (i) solitary males when males were captured alone (*n* = 21), (ii) as bachelor groups when adult males (*n* = 23) were captured as a group (*n* = 11 groups, average group size: 2.3±0.5) or (iii) as females (*n* = 9) when females were captured either alone (*n* = 4), with suckling cubs (*n_females_* = 2, *n_cubs_* = 4) or with weaned cubs (*n_females_* = 3, *n_cubs_* = 9). All cubs were excluded from the analysis. To test whether members of the same group (bachelor groups (*n* = 10)) are isotopically distinct from individuals in other groups, we performed an analysis of similarity (ANOSIM) using Primer 6 (Version 6-1-15; Primer-E Ltd.; [34]). Prior to analyses, unsigned data were square-root transformed to meet normal distribution. According to our analysis, group members were isotopcially similar. Therefore, we selected randomly one member of each group for further analysis.

#### Diet composition of free-ranging cheetahs

We assessed the relative contribution of the isotopic prey categories to the diet of individual cheetahs by applying a Bayesian isotope mixing model from the package SIAR version 4.1.3 [37] of the free statistical software R [38]). In these analyses, we distinguished between solitary males (*n* = 21), males as part of a bachelor group (*n* = 11) and females (*n* = 9). We excluded litter mate groups from the analysis because of small sample size for this category.

According to the isotopic information of captive cheetahs fed with donkey meat, we corrected the raw isotope data of free-ranging cheetahs for isotopic discrimination in ^15^N by subtracting 3.2‰ from raw δ^15^N values. We did not correct stable carbon isotope data, because there was no difference in δ^13^C between donkey and cheetah muscle tissue. Because the trophic discrimination established at AfriCat was based on muscle tissue and the samples from free-ranging cheetahs were RBC, we corrected the result for differences in stable isotope ratios between RBC and muscle tissue. For this we used published data on the closest phylogenetic relative, the red fox (*Vulpes vulpes*) [39] and added 1‰ to the stable nitrogen isotope ratios and 0.5‰ to the stable carbon isotope ratios.

To assess how sensitive the outcome of the Bayesian isotope mixing model is towards deviations in assumed discrimination factors, we repeated the model twice. In the first analysis, we used trophic discrimination factors as described by Roth and Hobson for red foxes (*Vulpes vulpes*) [39], i.e. we subtracted 0.7‰ from raw δ^13^C data and 2.6‰ from raw δ^15^N data. During the second analysis, we applied trophic discrimination factors as described by Parng and colleagues for felid species [40], i.e. we subtracted 5.5‰ from raw δ^13^C data and 4.1‰ from raw δ^15^N data.

### Dietary specialization of individual free-ranging cheetahs

For 9 individuals (8 males, 1 female) that were reported above, we assessed the dietary specialization of cheetahs by collecting four tissue samples. From each cheetah we collected a fur sample, muscle tissue, RBC and blood plasma. All samples were handled and stable isotope ratios were analyzed at the IZW as described above or in [26], [27], [41]. Raw stable isotope data was corrected for trophic discrimination by using discrimination factors established for the corresponding sample type in red fox, *Vulpes vupes* [39].

For description of data, we first tested if fur, tissue and blood samples differed in stable isotope ratios using a repeated measure ANOVA for each sample category, using the statistical software SYSTAT 13 (Systat Software Inc., Richmond, U.S.A.). We expected a larger variation in stable carbon isotope ratios in individual tissues than in stable nitrogen isotope ratios because of the higher contrast of potential prey species in stable carbon. We therefore performed the following analysis only with stable carbon isotope data. We calculated a Bray-Curtis similarity matrix and tested whether cheetahs were isotopically distinct according to an ANOSIM using Primer 6 (Version 6-1-15; Primer-E Ltd.; [34]). This was followed by a SIMPER analysis that quantifies the overall percentage dissimilarity between dyads of cheetahs and depicts the sample category which was most relevant in explaining the dissimilarity. To assess the degree of specialization of individual cheetahs, we plotted the frequency distribution of individual variance for both isotope data sets, assuming that low variances are indicative of a relatively high degree of specialization. Finally, we used the sample category most responsible for separating the cheetahs isotopically to conduct a variance component analysis. In this analysis, we assumed that in populations with highly specialized individuals within-individual variability is lower than between-individual variability, i.e. the majority of isotopic variance is explained by between-individual variation [30], [31].

## Results

### Isotopic data of potential prey

**Figure 1:**
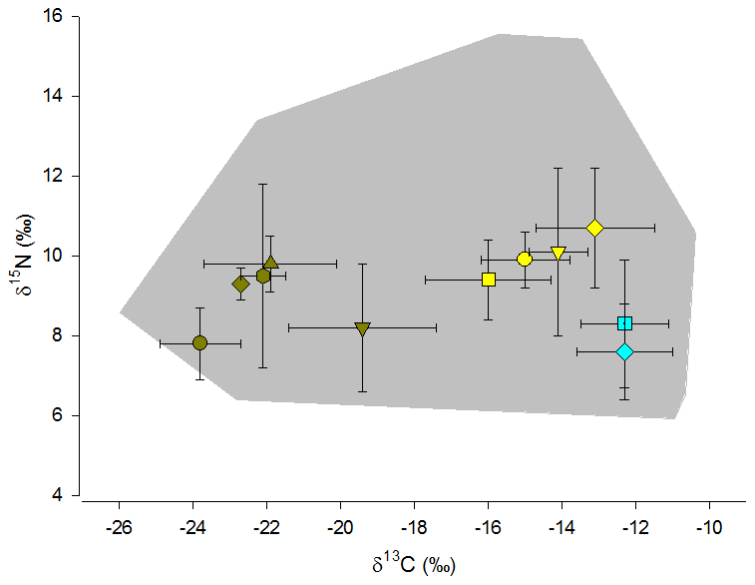
Nitrogen isotope ratios (δ^15^N; ‰; mean ± standard error) in relation to stable carbon isotope ratios (δ^13^C; **‰)** of the three prey categories. The grey zone indicates the minimum convex polgygon area that includes all individual data points of potential prey animals. Predominantly browsing prey species are labeled green (circle: steenbok; lower triangle: scrub hare; upper triangle: eland; polygon: springbok; diamond: kudu), predominantly grazing animals with high δ^15^N values are labeled yellow (diamond: springhare; lower triangle: gemsbok; circle: cattle, square: guinea fowl) and grazing animals with low δ^15^N values are labeled blue (warthog: diamond; hartebeest: square).

In total, we collected tissue material of 125 potential prey animals, comprising 11 species (Table 1). For stable carbon isotope ratios, we found a range of 15.6‰ between individual browsing (springbok: −26.0‰) and grazing herbivores (hartebeest: −10.4‰; Table S1). Stable nitrogen isotope ratios covered a range of 9.6‰ between potential prey individuals with low δ^15^N values (hartebeest: 5.9‰) and high δ^15^N values (gemsbok: 15.6‰; Table S1). A visual inspection of the bivariate graph plotting stable carbon and nitrogen isotope ratios of potential prey separates browsing and grazing individuals (Fig. 1). According to an analysis of similarity (ANOSIM), species differed in stable isotope ratios (global *R* = 0.572, *P* = 0.001). Posthoc tests and a similarity analysis differentiated between pairs (Table 2). Following the outcome of the SIMPER analyses (Table 2) and an evaluation of pair-wise differences, we defined three categories of prey species, namely browsing species (eland, kudu, springbok, steenbok and scrub hare), grazing animals with high δ^13^C values (gemsbok, cattle, springhare and guinea fowl) and grazing animals with low δ^13^C values (hartebeest and warthog). We pooled data of some species pairs even when pair-wise comparisons were significantly different, because the statistical separation seem to be driven by low variance and large sample size, yet differences were too small to warrant treatment as a separate isotopic prey category, e.g. in case of cattle and gemsbok.

**Table 1:** Mean stable carbon and nitrogen isotope ratios (± one standard deviation) of potential prey of cheetah in Namibian farmland.

Species:	N	δ^13^C (‰)	δ^15^N (‰)
***Grazer/high δ^15^N***		**−15.1±1.3**	**9.8±0.5**
Gemsbok (*Oryx gazella*)	31	−14.1±0.8	10.1±2.1
Cattle (Bos taurica)	28	−14.4±1.3	10.0±1.6
Guinea fowl (*Numida meleagris*)	4	−16.0±1.7	9.4±1.0
Springhare (*Pedetes capensis*)	10	−13.1±1.6	10.7±1.5
***Grazer/low δ^15^N***		**−12.3±1.3**	**8.0±1.4**
Hartebeest (*Alcelaphus buselaphus*)	15	−12.3±1.2	8.3±1.6
Warthog (*Phacochoerus africanus*)	14	−12.3±1.3	7.6±1.2
***Browser***		**−21.9±1.6**	**9.2±1.7**
Eland (*Taurotragus oryx*)	2	−22.7±0.1	9.3±0.4
Springbok (*Antidorcas marsupialis*)	7	−21.9±1.8	9.8±0.7
Kudu (*Tragelaphus strepsiceros*)	10	−22.1±0.6	9.5±2.3
Steenbok (*Raphicerus campestris*)	2	−23.8±1.1	7.8±0.9
Scrub hare (*Lepus saxatilis*)	3	−19.4±2.0	8.2±1.6

Mean values of prey categories were calculated on individual data from all species contributing to the specific category. The three categories used for analyses are highlighted in italic, bold letters.

**Table 2:** Dissimilarity matrix of potential prey species according to an analysis of similarity (ANOSIM).

**Eland**	0.99	**0.018**	0.91	**0.82**	0.995	**0.221**	**0.25**	0.995	0.995	**0.25**
	*P*<0.007	**P = 0.77**	*P*<0.002	***P*** **<0.067**	*P*<0.002	***P*** ** = 0.25**	***P*** ** = 0.33**	*P*<0.008	*P*<0.015	***P*** ** = 0.20**
10.9	**Harte-**	0.988	0.343	0.406	0.652	0.978	0.995	**0.0**	**0.261**	0.824
C (81.5)	**beest**	*P*<0.001	*P*<0.002	*P*<0.015	*P*<0.001	*P*<0.001	*P*<0.001	***P*** ** = 0.42**	***P*** ** = 0.80**	*P*<0.001
**2.6**	11.3	**Kudu**	0.952	0.817	0.981	**0.045**	**0.065**	0.988	0.974	**0.226**
**N (82.5)**	C (75.1)		*P*<0.001	*P*<0.002	*P*<0.001	***P*** ** = 0.231**	***P*** ** = 0.333**	*P*<0.001	*P*<0.01	***P*** ** = 0.12**
8.3)	4.9	9.3)	**Gemsbok**	**0.134**	0.108	0.878	0.984	0.5	0.192	0.792
C (83.0	N (60.1)	C (70.2		***P*** ** = 0.219**	*P* = 0.001	*P*<0.001	*P*<0.002	*P*<0.001	P = 0.03	*P* = 0.002
6.0	5.9	7.1	**3.6**	**Guinea**	**0.185**	0.857	0.960	0.689	**0.21**	**0.537**
C (85.6)	C (61.4)	C (67.5)	**C (50.2)**	**Fowl**	***P*** ** = 0.124**	*P*<0.003	*P* = 0.007	*P*<0.003	***P*** ** = 0.067**	***P*** ** = 0.086**
6.9 C	5.3	7.8	3.0	2.6	**Cattle**	0.958	1.0	0.791	0.389	0.958
(87.4)	(51.7)	C (71.8)	N (58.5)	C (54.1)		*P*<0.001	*P*<0.002	*P*<0.001	*P*<0.001	*P*<0.001
**1.9**	10.6	**3.0**	8.0	5.8	6.4)	**Spring-**	**0.539**	0.993	0.928	**0.361**
**N (59.4)**	C (78.2)	**N (69.3)**	C (79.6)	C (80.8)	C (86.4)	**bok**	***P*** ** = 0.083**	*P*<0.001	*P*<0.001	***P*** ** = 0.10**
**2.4**	11.5	**3.6**	10.4	7.9	9.3	3.8	**Steen-**	0.997	1.0	**0.333**
**N (69.6)**	C (85.1)	**N (68.5)**	C (74.8)	C (76.6)	C (74.0)	N (56.3)	**bok**	*P*<0.008	*P*<0.015	***P*** ** = 0.30**
11.2	**3.8**	11.6	5.5	6.3	6.0	11.2	11.3	**Warthog**	0.522	0.896
C (80.2)	**N (59.4)**	C (74.2)	N (60.0)	C (59.9)	C (59.9)	C (75.3)	C (87.4)		*P*<0.001	*P*<0.001
9.4	**5.1**	10.1	3.8	**4.6**	3.7)	8.8	11.9	5.7	**Spring-**	0.879
C (82.9)	**N (65.2)**	C (73.5)	N (58.1)	**C (59.1)**	C (57.5)	C (82.8)	C (72.7)	N (69.6)	**hare**	*P*<0.003
**4.2**	8.6	8.6	7.2	**5.0**	6.0	**4.0**	**4.7**	8.5	8.6)	**Scrub-**
**C (59.3)**	C (75.8)	C (75.8)	C (63.3)	**C (60.0)**	C (61.9)	**N (51.4)**	**C (68.6)**	C (77.5)	C (64.1)	**-hare**

Upper numbers in cells of the lower triangle of the matrix depict the percentage dissimilarity of pair-wise comparisons according to a SIMPER analysis based on stable carbon and nitrogen isotope data. The letter and numbers in the lower part of the same cell depict the element which is most responsible for dissimilarity and the percentage of contribution of this specific element for explaining dissimilarity between pairs. Numbers in the upper triangle of the matrix depict the R- and P-value of pair-wise comparisons according to an ANOSIM. R-values range between 0 and 1 with values above 0.75 indicating separation of species pairs and values below 0.25 as barely separable species based on stable isotope ratios [34]. Pairs of species with similar stable isotope signature are highlighted in bold (see text for exemptions) and prey categories are highlighted with colours: browser in green, grazer/high δ^15^N in yellow and grazer/low δ^15^N in blue.

### Diet of cheetahs on Namibian farmland

#### Isotopic discrimination in cheetah tissue

δ^13^C values of cheetah muscle of nine captive cheetahs at AfriCat averaged −15.6±0.9‰. This value did not differ from the mean δ^13^C of donkey meat (−15.7±1.4‰, Mann-Whitney U-Test, *U* = 28, *n_1_* = 9, *n_2_* = 7, *P* = 0.71) that was fed to cheetahs over 2 months prior to tissue collection. In contrast, δ^15^N values of cheetah muscle averaged 11.6±0.6‰, which was higher than the mean δ^15^N of donkey meat (8.4±0.7‰; *U* = 0, *P*<0.002). Thus, muscle tissue of cheetahs was enriched in ^15^N in relation to that of the diet by 3.2‰.

#### Diet of free-ranging cheetahs

**Figure 2:**
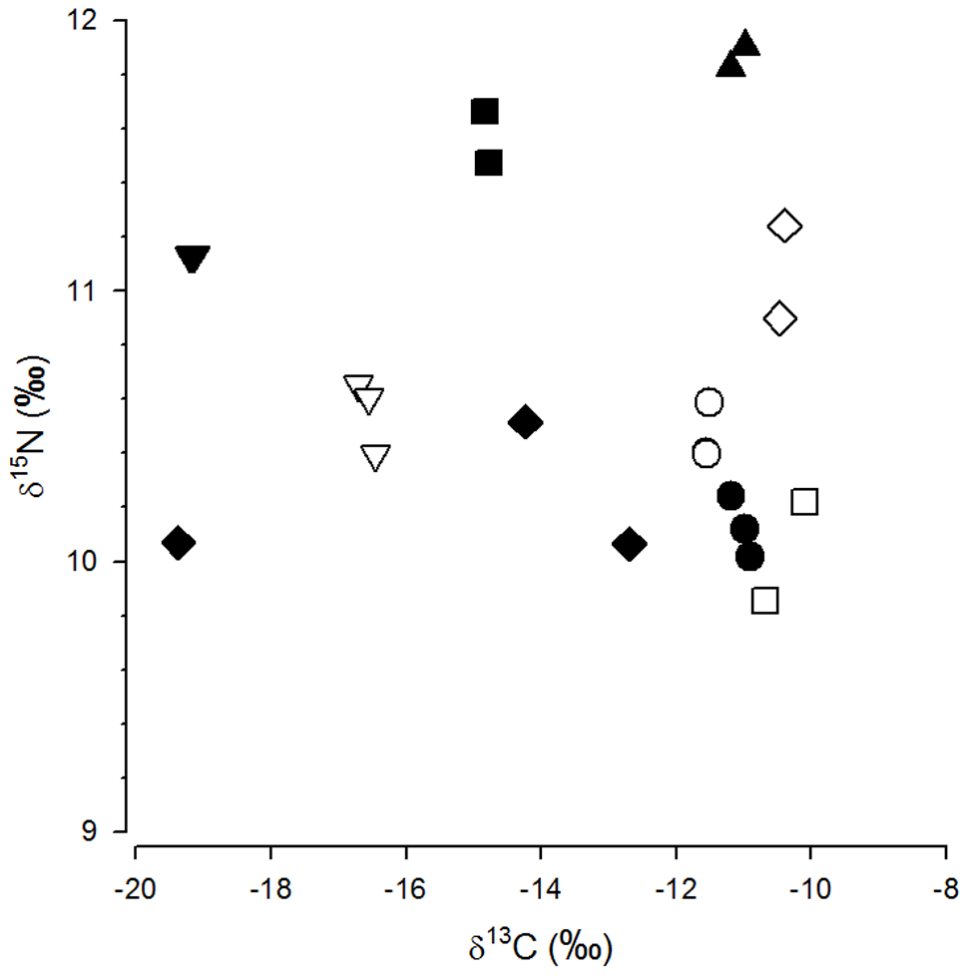
Nitrogen isotope ratios (δ^15^N; ‰) in relation to stable carbon isotope ratios (δ^13^C; ‰) in cheetah groups of bachelor males. Each symbol represents one individual. Groups are indicated by different symbols.

Members of different groups differed in their isotopic composition (ANOSIM: global *R* = 0.814, *P*<0.001; Fig. 2). For further analysis, we included only a randomly selected member of each group. Accordingly, we reduced the data set of male cheetahs living in groups to 10 representatives, resulting in a total data set of 40 cheetahs, i.e. 21 solitary males, 10 males of bachelor groups and 9 females. Stable isotope ratios of these cheetahs averaged −15.8±3.2‰ for carbon (range −10.3‰ to −20.7‰) and 11.0±0.9‰ for nitrogen (range: 9.5‰ to 13.3‰) (Table S2).

**Figure 3:**
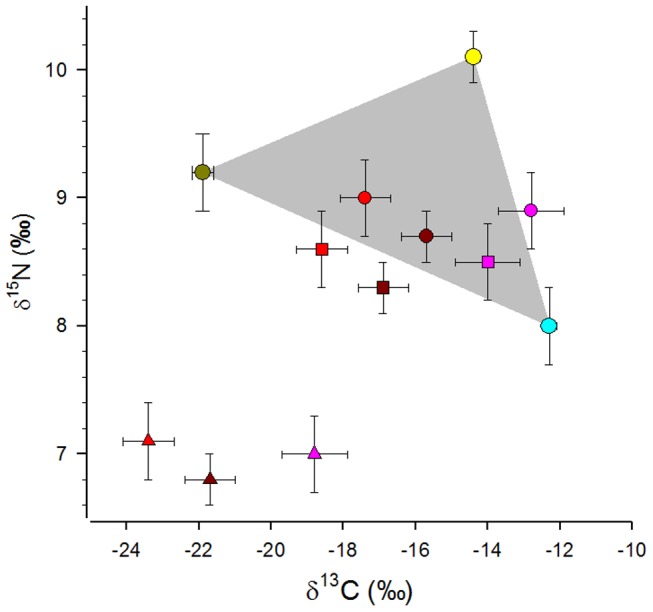
Nitrogen isotope ratios (δ^15^N; mean ± standard error, ‰) in relation to stable carbon isotope ratios (δ^13^C; ‰) in the three prey categories (browser: green circle, grazers with high δ^15^N: yellow; grazers with low δ^15^N: blue) and in three categories of cheetahs (note that isotopic data of cheetahs were corrected for trophic discrimination): females (red symbols), solitary males (dark red) and males in bachelor groups (pink). The grey zone indicates the isoscape covered by the mean values of prey categories. We assessed the sensitivity of the model output based on 3 scenarios with suggested trophic discriminations. (1) trophic discrimination established by this study (circles), (2) by Roth and Hobson [39] (squares) and (3) by Parng and coauthors [40] for felid species (triangles).

According to an ANOSIM, cheetah categories differed in isotopic composition (global *R* = 0.115, *P* = 0.026), i.e. males of bachelor groups were statistically different in isotopic composition from solitary (*R* = 0.148, *P* = 0.022) and from females (*R* = 0.444, *P* = 0.004), whereas solitary males and females were isotopically more similar (*R* = −0.092, *P* = 0.876). δ^13^C values averaged −16.2±3.1‰ for solitary males, −13.3±2.8‰ for males in bachelor groups and −17.9±2.0‰ for females (either as mothers with cubs or as solitary females). δ^15^N values averaged 10.9±0.9‰ for solitary males, 11.1±0.9‰ for males in bachelor groups and 11.2±0.9‰ for females. Isotopic data that was corrected for trophic discrimination was within the boundaries of the isospace of the potential prey categories, except for those of males in bachelor groups, which were slightly adjacent to the minimum convex polygon between mean values, but not outside the minimum convex polygon of isotopic data of individual data points of potential prey animals (Fig. 1 and 3).

**Figure 4:**
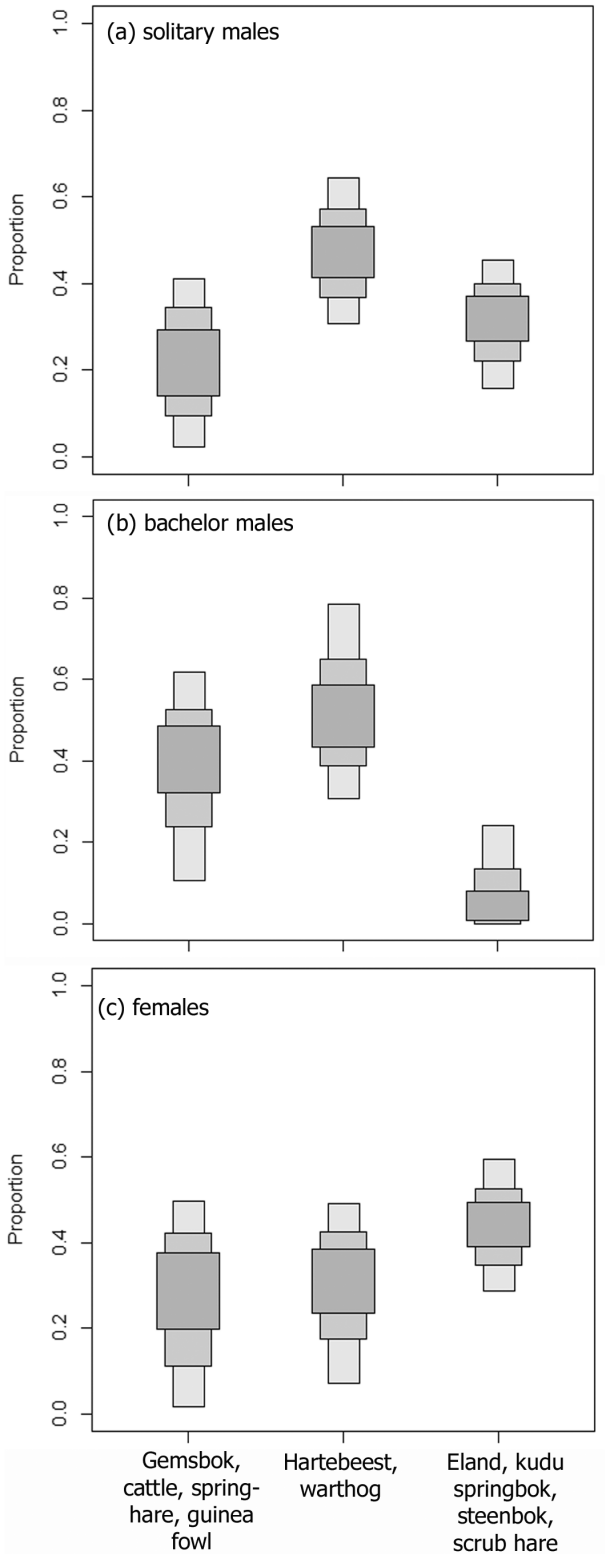
Relative proportions of isotopically distinct categories of potential prey to the diet of solitary male cheetahs (a), male cheetahs foraging in a bachelor group (b) and female cheetahs (c) as determined by a Bayesian isotopic mixing model. Box plots show the relative proportions for each food source with 95% (dark grey), 75%, 25% (medium grey) and 5% (light grey) confidence intervals.

According to the Bayesian mixing model, solitary males fed mostly on hartebeest and warthog (mode percentage contribution: 47.4%; lower 95% confidence interval: 30.4%; upper 95% CI: 63.9%), followed by browsers, including eland and kudu as valuable trophy species (mode PC: 31.9%; lower 95% CI: 15.5%; upper 95% CI: 45.9%) and to a lesser extent on grazers with high δ^15^N values, including cattle and gemsbok as a valuable trophy species (mode PC: 17.9%; lower 95% CI: 1.9%; upper 95% CI: 40.5%; Fig. 4). The model suggested that males of bachelor groups fed mostly on hartebeest and warthog (mode PC: 50.2%; lower 95% CI: 30.1%; upper 95% CI: 78.6%), followed by grazers with high δ^15^N values (mode PC: 40.3%; lower 95% CI: 10.7%; upper 95% CI: 60.6%) and to a lesser extent on browsers (mode PC: 3.4%; lower 95% CI: 0.0%; upper 95% CI: 23.9%; Fig. 4). Finally, female cheetahs were suggested to fed predominantly on browsers (mode PC: 45.9%; lower 95% CI: 29.2%; upper 95% CI: 59.8%), followed by grazers with low δ^15^N values (mode PC: 33.0%; lower 95% CI: 7.0%; upper 95% CI: 49.6%) and by grazers with high δ^15^N values (mode PC: 31.2%; lower 95% CI: 1.8%; upper 95% CI: 49.4%; Fig. 4).

In our sensitivity analysis we investigated the effect of assumed trophic discrimination factors on the output of the Bayesian mixing model. When using trophic discrimination factors as suggested by [39], mean values of the three cheetah categories where inside or adjacent to the isoscape area of the potential prey species (Fig. 3). Similar to the previous model that was based on our own established discrimination factors, the model suggested that solitary males fed mostly on hartebeest and warthog (mode PC: 49.7%; lower 95% CI: 33.8% upper 95% CI: 63.2%), followed by browsers (mode PC: 41.7%; lower 95% CI: 29.2%; upper 95% CI: 56.0%) and to a lesser extent on grazers with high δ^15^N values (mode PC: 3.1%; lower 95% CI: 0.0%; upper 95% CI: 21.6%). The model suggested that males in bachelor groups consumed mostly hartebeest and warthog (mode PC: 62.2%; lower 95% CI: 35.2%; upper 95% CI: 80.1%), followed by grazers with high δ^15^N values (mode PC: 26.4%; lower 95% CI: 2.1%; upper 95% CI: 49.7%) and to a lesser extent on browsers (mode PC: 13.2%; lower 95% CI: 0.2%; upper 95% CI: 31.4%). Finally, female cheetahs were suggested to feed predominantly on browsers (mode PC: 58.7%; lower 95% CI: 38.2%; upper 95% CI: 72.3%), followed by grazers with low δ^15^N values (mode PC: 29.2%; lower 95% CI: 3.9%; upper 95% CI: 47.8%) and to a low extent on grazers with high δ^15^N values (mode PC: 6.1%; lower 95%CI: 0.0%; upper 95% CI: 39.0%). We did not calculate a mixing model for raw data corrected according to the discrimination factors suggested for felid species by Parng and colleagues [40], because mean values of corrected cheetah data were well outside the minimum convex polygon of mean isotopic data of prey categories (Fig. 3).

### Dietary specialization of individual free-ranging cheetahs

**Figure 5:**
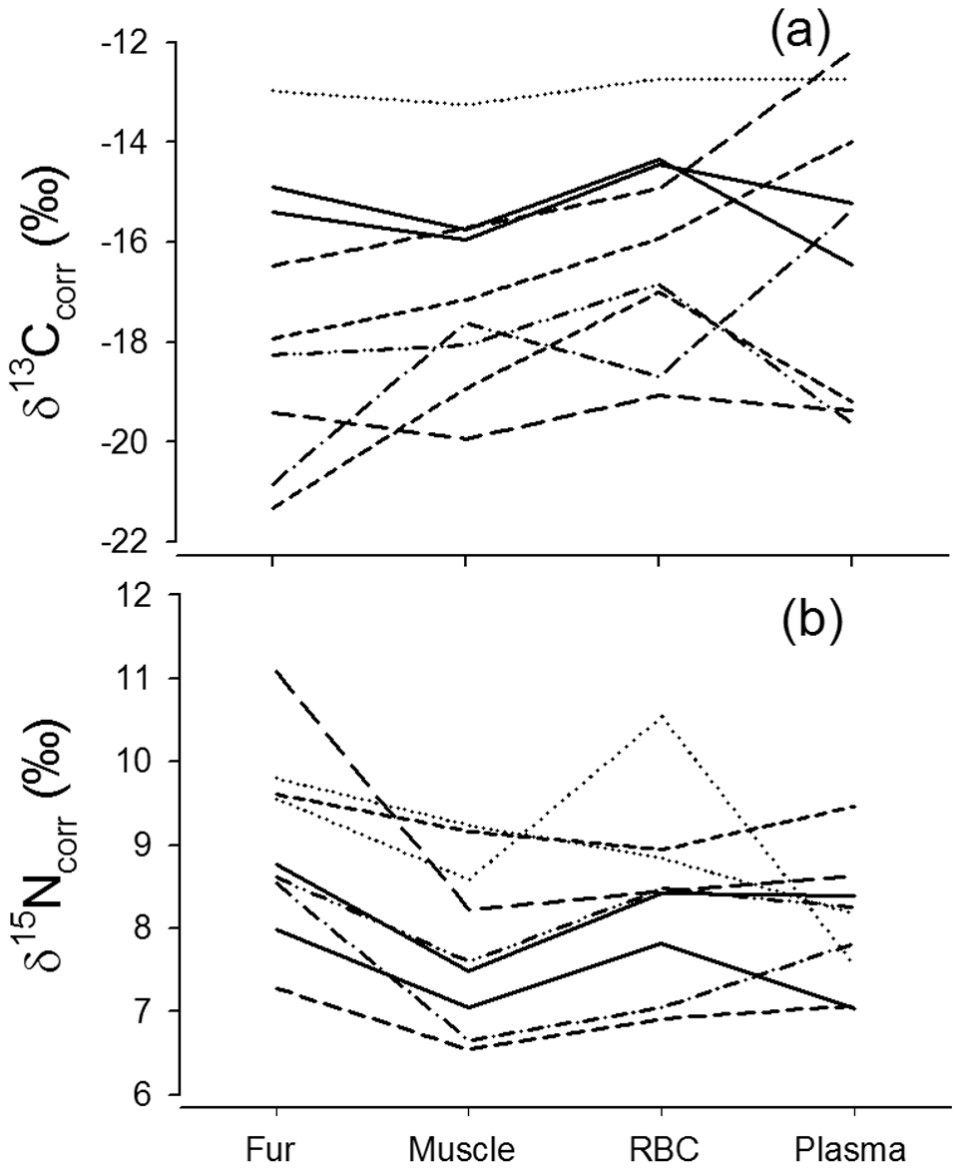
Stable carbon isotope ratios (a) and nitrogen isotope ratios (b) in 9 cheetahs (8 males, 1 female) in four tissues with decreasing isotopic retention time: fur, muscle, red blood cells (RBC) and plasma. Values of the same individual are connected with individually identifiable lines.

**Figure 6:**
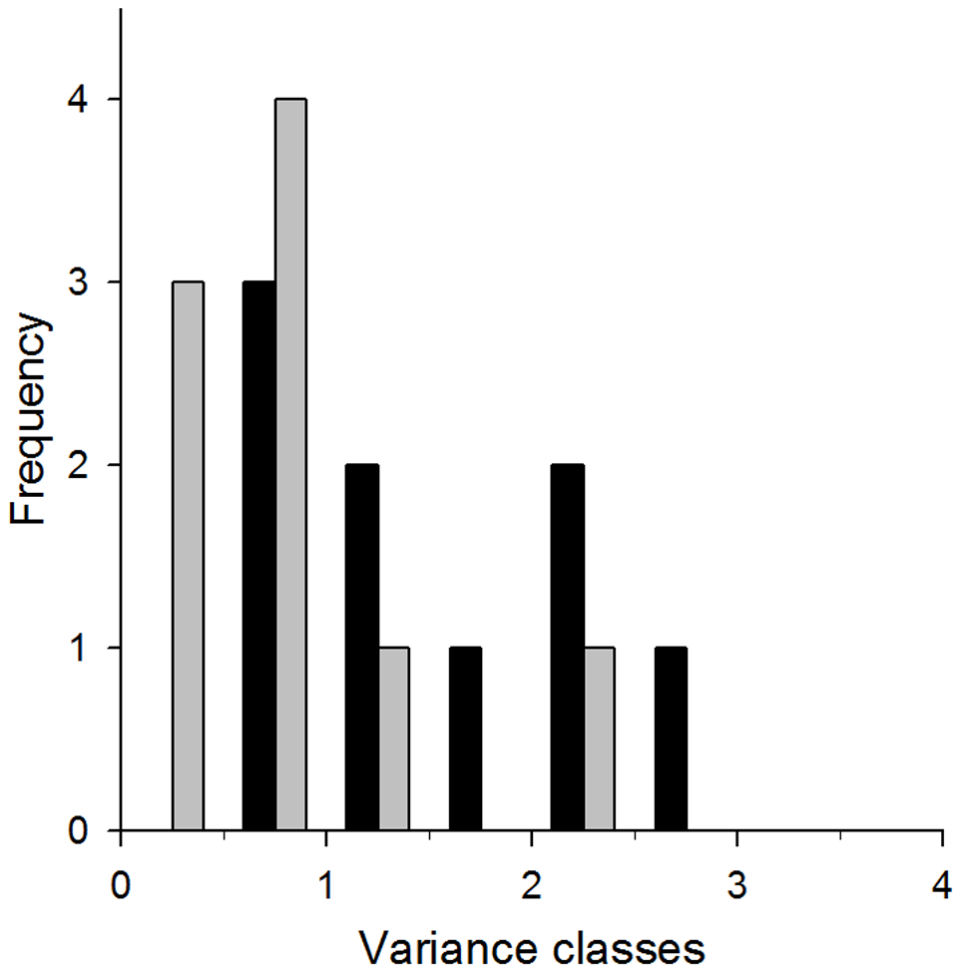
Frequency distribution of within-individual variances for stable carbon isotope ratios (δ^13^C; black bars) and nitrogen isotope ratios (δ^15^N; grey bars).

A repeated measures ANOVA revealed that fur, muscle, RBC and blood plasma differed in δ^13^C values corrected for trophic discrimination (*F_3,35_* = 3.17; *P* = 0.042; Fig. 5), yet posthoc Tukey Kramer tests revealed non of the pair-wise comparison to be significant (*P*>0.05). δ^15^N values corrected for trophic discrimination also differed among tissue types (*F_3,35_* = 6.26; *P*<0.003; Fig. 5), but posthoc Tukey Kramer tests for multiple comparisons showed that only δ^15^N values of fur and muscle (*Q* = 5.7, *P*<0.01) and those of fur and plasma (*Q* = 4.7, *P*<0.05) differed. All other pair-wise comparisons proved not to be significant. Averaged δ^13^C_corr_ values of individual cheetahs ranged from −21.3‰ to −12.2‰ and δ^15^N_corr_ values from 6.5‰ to 11.1‰ (Table S3). According to an ANOSIM, cheetah individuals were isotopically distinct (global *R* = 0.516, *P*<0.001). Twenty one out of 36 possible dyadic comparisons of stable isotope ratios, i.e. 58% of all possible comparisons were significantly different (Table 3 in File S1). Dissimilarities among dyads of cheetahs were almost always best explained by variation in δ^13^C values, indicating that inclusion of varying proportions of browsers or grazers in cheetah diets are most likely responsible for the isotopic specialization of cheetahs. None of the cheetah individuals showed a unique isotopic signature not shared by any of the other cheetahs. Furthermore, we looked at the variance/covariance patterns of intra-individual isotopic values. The frequency distribution of within-individual variances of stable isotope data were left skewed (Fig. 6) and individual variances of δ^13^C and δ^15^N values were correlated (*d.f.* = 8, *R^2^* = 0.679, *P*<0.05). To assess the extent of specialization based on a variance component analysis, we focused on δ^13^C values since they were most responsible for differences among individuals (Table 3). According to this analysis, between-individual variation explained 75% of the variation in δ^13^C values and within-individual variation 25%.

**Table 3:** Stable carbon and nitrogen isotope ratios (mean ± one standard deviation) corrected for trophic discrimination for 9 cheetahs (8 males (M), 1 female (F)) and matrix of dissimilarity.

δ^13^C_corr_ (‰)	δ^15^N_corr_ (‰)	Dyadic comparisons								
−15.3±0.6	7.5±0.5	**M1**	**0.510**	**0.781**	**0.99**	**0.531**	−0.094	−0.177	**0.563**	**1.0**
			***P*** ** = 0.03**	***P*** ** = 0.03**	***P*** ** = 0.03**	***P*** ** = 0.03**	*P* = 0.743	*P* = 0.800	***P*** ** = 0.03**	***P*** ** = 0.03**
−16.2±1.7	9.0±0.7	**2.8**	**M2**	−0.031	**0.76**	0.031	0.292	0.448	**0.813**	**0.573**
		**N (55.0)**		*P* = 0.371	***P*** ** = 0.03**	*P* = 0.429	*P* = 0.171	*P* = 0.086	***P*** ** = 0.03**	***P*** ** = 0.03**
−18.2±1.1	9.3±0.3	**4.3**	2.4)	**M3**	0.292	−0.208	**0.667**	**0.635**	**0.99**	0.01
		**C (58.6)**	C (67.6)		*P* = 0.057	*P* = 0.943	***P*** ** = 0.03**	***P*** ** = 0.03**	***P*** ** = 0.03**	*P* = 0.43
−19.4±0.4	8.2±0.4	**4.4**	**3.6)**	2.5	**M4**	0.042	**0.792**	**0.771**	**1.0**	−0.094
		**C (80.8)**	**C (73.6)**	C (53.5)		*P* = 0.314	***P*** ** = 0.03**	***P*** ** = 0.03**	***P*** ** = 0.03**	*P* = 0.714
−18.1±2.3	9.1±1.3	**4.1**	2.9	2.5	2.5	**F5**	0.448	0.512	**0.917**	−0.904
		**C (60.3)**	C (61.7)	C (52.4)	C (52.9)		*P* = 0.057	*P* = 0.057	***P*** ** = 0.03**	*P* = 0.714
−14.8±1.9	7.5±0.8	2.2	3.3	**4.8**	**5.2**	4.7	**M6**	−0.177	0.292	**0.854**
		C (50.7)	N (50.6)	**C (61.1)**	**C (77.3)**	C (61.2)		*P* = 0.829	*P* = 0.171	***P*** ** = 0.03**
−15.4±0.9	7.0±0.3	**2.0**	3.5	**4.8**	**4.9**	4.7)	2.7	**M7**	**0.542**	**0.854**
		**C (59.4)**	N (60.1)	**C (51.4)**	**C (72.1)**	C (53.9)	C (53.6)		***P*** ** = 0.03**	***P*** ** = 0.03**
−12.9±0.2	8.2±0.6	**3.3**	**4.2**	**6.0**	**6.6**	**6.1**	3.5	**3.9**	**M8**	**1.0**
		**C (70.7)**	**C (75.9)**	**C (79.9)**	**C (89.3)**	**C (79.0)**	C (62.0)	**C (62.0)**		***P*** ** = 0.03**
−19.1±1.8	9.1±1.3	**4.9**	**3.0)**	1.9	1.7	2.3)	**5.3**	**5.4**	**6.6**	**M9**
		**C (67.7**)	**C (77.9)**	C (62.2)	N (54.7)	C (53.1)	**C (69.7)**	**C (60.3)**	**C (84.9)**	

Upper numbers in cells of the lower triangle of the matrix depict the percentage dissimilarity of pair-wise comparisons according to a SIMPER analysis based on replicate stable isotope ratios. The letter and numbers in the lower part of the same cell depict the element which is most responsible for dissimilarity and the percentage of contribution of this specific element for explaining dissimilarity between pairs. Numbers in the upper triangle of the matrix depict the R- and P-value of pair-wise comparisons according to an ANOSIM. R-values range between 0 and 1 with values above 0.75 indicating separation of species pairs and values below 0.25 as barely separable species based on stable isotope ratios [34]. Pairs of cheetahs with significantly distinct stable isotope signature are highlighted in bold.

## Discussion

We studied the diet of cheetahs on Namibian farmland to answer the following questions: Which prey species do cheetahs prefer in the anthropogenically influenced landscape of southern Africa? In particular, do cheetahs hunt largely on livestock such as cattle and on valuable trophy species such as eland, kudu and gemsbok? And, are individual cheetahs specialists, hunting preferably on one or a few prey categories? We used a stable isotope approach for answering these questions, because previous studies on African carnivores demonstrated the significance of such an approach [42]. Stable isotopes are particularly useful to our study goals, because stable carbon and nitrogen isotopes separate most of the potential prey according to their membership to either a C3 or C4 food web and according to their enrichment in ^15^N in relation to ^14^N. Also as endogenous markers, isotopic signatures integrate over the period of tissue growth [14]. Therefore, it is possible to obtain insights into temporal aspects of feeding behavior. Using stable isotope data, we were able to distinguish between three isotopic prey categories. A Bayesian mixing model suggested that cheetahs consumed members of all prey categories, albeit to varying extents. None of the three cheetah categories of solitary males, males foraging as a group of bachelors and females fed predominantly on the prey category that included cattle and gemsbok. Both solitary and bachelor males fed mostly on grazers with low δ^15^N values, i.e. hartebeest and warthogs. In contrast, female cheetahs fed predominantly on browsers. We assume that they prey largely on small browsing ungulates, such as steenbok or springbok given the smaller size of female cheetahs compared to male cheetahs [43], and not so much on calves of valuable trophy species such as eland and kudu that are likely to be defended by their mothers. Finally, we assessed the degree of specialization in individual cheetahs and found that even though no individual was isotopically unique, most of them showed a relatively high degree of specialization.

### Isotopic data of potential prey

As a prerequisite of our study, we depended on isotopically distinct prey categories. Our analysis separated three prey categories. We defined one category as grazers with high δ^15^N values that included grazing herbivores (gemsbok and springhare), guinea fowls that presumably fed on grass seeds and grass-processing insects, and cattle. The second category consisted of grazers with low δ^15^N, i.e. warthog and hartebeest. The third prey category consisted of members of a C3 food web, namely browsing herbivores such as eland, kudu, springbok, steenbok and scrub hare. Our categorization of species into prey categories was justified by statistical separation and overall small isotopic differences between species pairs that made a separation questionable even in light of a significant outcome of statistical tests. In some cases, statistical detection of relatively small differences was facilitated by relatively large sample sizes, e.g. in the comparison of isotopic data from gemsbok-cattle or gemsbok/cattle-springhare. In some other cases, isotopic data were not distinguishable according to the SIMPER analyses, yet the inability to detect a difference may have originated from low sample sizes and relatively large variation of data, e.g. eland-guinea fowl or scrub hare-guinea fowl pairs. In these cases, we found it justified to pool or separate species pairs accordingly.

Stable isotope data of ungulate species of this study were similar to those reported before for free-ranging herbivores in southern Africa [21]–[27]. Yet, two assumptions of this study must be explicitly recognized. Firstly, we assumed that stable isotope signature of plants and prey species do not vary largely between seasons and years. Previous studies in Southern Africa indicated that plants and even herbivore consumers may vary to some extent in stable isotope composition according to precipitation patterns and related shifts in isotopic baselines of ecosystems [26]. We assume that such fluctuations had no large effect on the outcome of our study, because of the small magnitude of temporal fluctuations in isotopic composition of herbivore tissues or plant matter in relation to the large isotopic contrasts in the reported prey categories [26], [27]. Secondly, we missed a few potential prey species of cheetahs at our study site. For duiker we assumed that they fall into the category of browsers because of their preference for browse [23]. Even though we may have not included all potential prey species of wild cheetahs in our study, we suggest that the outcome of our mixing models may not change drastically with the inclusion of further species, since the isotopic space, as shown in figure 1, is covering almost all isotopic niches of potential prey species for cheetahs. However, we suggest being cautious in the identification of species as the preferred or non-preferred prey, because some potential prey species were missing in this study, and because isotopic prey categories subsume several species. Also, the overall isospace of the potential prey species, indicated by the minimum convex polygon on the bivariate plot of figure 1, suggests that stable isotope ratios varied largely in potential prey species. Lastly, we acknowledge that in underdetermined mixing scenarios, i.e. those were number of sources are higher than the number of explanatory variables (i.e. isotopes), rare solutions may be as likely as the most frequent ones [44].

### Diet of cheetahs on Namibian farmland

In our feeding experiment with captive cheetahs, we found that muscle tissues were enriched in ^15^N in relation to ^14^N by 3.2‰ when compared to donkey meat, yet we did not measure an enrichment of ^13^C in relation to ^12^C in muscle tissue of cheetahs compared to their diet. The discrimination of heavy nitrogen in relation to light nitrogen is similar to what previous studies reported for carnivore species [39], [45]. However, Roth and Hobson [39] documented a small, yet significant discrimination of ^13^C between carnivore tissue and prey tissue. The discrepancy in discrimination factors between their and our findings may be partly explained by the fact that study animals consumed food items that differed in macronutrient composition. The macronutrient composition of food can influence the trophic discrimination of stable carbon and nitrogen isotopes in carnivores [46]. Whereas cheetahs in our study were only fed with entire animals or animal parts, red foxes in the other study were fed with commercial food pellets that may differ from meat in nutrient composition. If pellets include for example a large proportion of carbohydrates of plant origin, isotopic discrimination may be affected by selective routing of carbon-rich nutrients to, for example, the pool of oxidative fuel [47]. The meta-analysis of Caut and colleagues [45] suggests an isotopic discrimination of ^13^C in relation to ^12^C between mammal consumers and their diet of on average 0.5‰. Codron and colleagues [42] reported an isotopic discrimination of ^13^C in relation to ^12^C of 2.6‰ between diet and carnivore hair. A larger sample size in our study may have revealed a subtle discrimination of stable carbon isotopes, yet given the presumed small magnitude of this discrimination, we suggest that this has no large effect on the outcome of our mixing models. Indeed, our first sensitivity analysis based on the trophic discrimination measured for *Vulpes vulpes* [39] revealed that the outcome of the mixing model does not change substantially. This finding suggests that our mixing model is robust towards small deviations in measured and assumed discrimination factors.

A recent study suggested larger discrimination factors for both carbon and nitrogen in fur of felid species [40]. The suggested trophic discrimination of ^13^C in relation to ^12^C of about 5.5‰ for felid species contrasts with our and other studies [39], [42], yet the authors also demonstrate a lack of trophic discrimination for stable carbon isotopes in a single lion individual [40]. Trophic discrimination of nitrogen isotopes was reported as 4.1‰ between felid fur and diet. Overall, data of Parng and colleagues [40] is very heterogenous among the four studied felid species, which could be partly explained by low sample sizes, ranging between 1 and 3 individuals, by the consumption of industrial food pellets, and also by the medical treatment of felids during the study period. Correcting the raw isotope data of cheetahs in our study by using the discrimination factors as suggested by Parng and colleagues [40] leads to very low values, i.e. an average δ^15^N of 6.9±0.9‰ and an average δ^13^C of −21.3±3.2‰. The corrected nitrogen isotope values are substantially lower than any of the prey isotope data (Table 1, Fig. 3). Because the corrected mean values were well outside the isoscape of our potential prey species, we considered the discrimination factors by Parng and coauthors [40] as not relevant for cheetahs.

In general, solitary and bachelor males prefer hartebeest and warthogs over other prey categories. Furthermore, solitary males and females exhibited relatively low δ^13^C values, which is indicative of a large proportion of prey species from a C3 food web in the diet. Our mixing model supports this notion by highlighting that female cheetahs fed mostly and solitary males fed second mostly on browsers. We assume that female cheetahs hunt on smaller browsing species such as steenbok, springbok and not very often on calves of the larger browsing trophy species such as eland or kudu, whereas solitary males might also hunt on calves of larger browsing species [9], [10], [27]. However, we can not quantify any prey sizes the cheetahs might kill. The mode proportion contribution of grazers with high δ^15^N values, including cattle and gemsbok, in the diet of solitary males was 17.9%, in males in bachelor groups 40.3% and in female cheetahs 31.2%. Yet, the lower 95% CI was low in solitary males (1.9%) and in female cheetahs (1.8%), questioning the relevance of this prey category in the diet of these cheetah categories. Male cheetahs foraging in bachelor groups exhibited a lower 95% CI value of about 10.7%, which suggested that they, indeed, include some grazers with high δ^15^N values in their diet. However, because we pooled data of four prey species to this prey category, we can not tell to what extent bachelor males included cattle and gemsbok in their diet. Considering all cheetahs of our study, grazers with high δ^15^N values do not constitute a major part of cheetah diet on Namibian farmland, a notion that is also supported by conventional analyses of cheetah feces [11], [12].

### Dietary specialization of individual free-ranging cheetahs

We based our assessment of individual dietary specialization on replicate measurements of stable isotope ratios in four tissues of 9 cheetah individuals. Variation of stable isotope ratios within cheetah individuals was low, yet none of the cheetahs exhibited a unique isotopic signature that differed from all other individuals. This suggests that individual cheetahs overlapped at least partly in their diet. Furthermore, variance/covariance patterns and variance component analysis revealed that most of the individuals were isotopic specialists, i.e. it is very likely that the majority of cheetahs hunted a specific isotopic prey category. Previous conventional and isotopic studies have already pointed out that many mammalian predators, e.g. cheetahs, brown hyenas (*Hyaena brunnea*), wolves (*Canis lupus*) and sea otters (*Enhydra lutris*) are individual dietary specialists [30], [48]–[51], yet this aspect of predator behavior has never been studied before in a felid species using stable isotopes. We suggested two biological scenarios for the development of individual hunting tactics: a patchy distribution of isotopically contrasting prey species or sex- or individual-specific hunting behaviors. On Namibian farmland, prey animals are largely philopatric because of the perennial availability of water from artificial waterholes [52], [53]. Therefore, for our study area we assume it to be unlikely that isotopic specialization of cheetahs is a result of patchy distribution of prey species that forced cheetah to specialize on particular prey species or isotopic prey categories. Cheetah males have been reported to develop techniques to overcome particular prey animals, and males and females to hunt on different prey sizes [9]–[11], [49]. This suggests that sex- and individual-specific hunting behaviours are the underlying factors for isotopic specialization in cheetahs.

## Conclusions

We investigated the diet composition of free-ranging cheetahs on Namibian farmland, the area in Africa that harbors the largest population of cheetahs. We designed our study to elucidate which category of prey species cheetahs are hunting and whether they are specialists on certain isotopic prey categories. Also, we were interested whether cheetahs hunt largely on livestock and/or on valuable trophy species. The latter question is most relevant for formulating management guidelines to conserve populations of Namibian cheetahs. Although our findings revealed a low percentage of cattle and valuable trophy species in the diet of the cheetahs, the monetary losses for farmer still might be substantial. The loss of a single cattle calves may convert to approximately 550 US$ for a Namibian farmer and the loss of a calf that might have grown to an adult trophy animal might convert to approximately 1,900 US$, 1,400 US$ and 650 US$ for eland, kudu and gemsbok respectively. A conversion of our findings into actual numbers of individual losses per species might be useful in further studies, yet this would require an estimation of total number of consumed prey individuals per cheetah, which was not part of our study.

## Supporting Information

Table S1 **Raw isotopic data of potential prey species.**
(DOC)

Table S2 **Raw isotopic data of cheetahs (1 = solitary males, 2 = males of bachelor groups, 3 = females.**
(DOC)

Table S3 **Raw isotopic data of 4 tissues in 9 cheetahs (1 = female, 8 males), rbc = red blood cells.**
(DOC)
