# Supplementary material for: The Conflict between Cheetahs and Humans on Namibian Farmland Elucidated by Stable Isotope Diet Analysis
Source: PLoS One. 2014 Aug 27;9(8):e101917. doi: 10.1371/journal.pone.0101917 (PMC4146470; doi:10.1371/journal.pone.0101917)
Supplement: Table S1 — Raw isotopic data of potential prey species. (DOC) [file pone.0101917.s001.doc]

Table S1:

| **Species** | **δ^13^C (‰)** | **δ^15^N (‰)** |
| --- | --- | --- |
| *Cattle* (*Bos taurica*) | | |
| 1 | -15.5 | 8.3 |
| 2 | -15.0 | 9.5 |
| 3 | -13.6 | 9.7 |
| 4 | -13.1 | 8.5 |
| 5 | -16.3 | 10.6 |
| 6 | -16.3 | 10.0 |
| 7 | -16.6 | 10.4 |
| 8 | -15.3 | 9.8 |
| 9 | -12.6 | 10.2 |
| 10 | -14.8 | 10.0 |
| 11 | -17.3 | 9.8 |
| 12 | -14.8 | 8.9 |
| 13 | -14.6 | 10.8 |
| 14 | -17.0 | 11.0 |
| 15 | -16.6 | 10.7 |
| 16 | -14.2 | 9.5 |
| 17 | -15.0 | 10.8 |
| 18 | -14.7 | 9.5 |
| 19 | -14.1 | 10.8 |
| 20 | -14.2 | 9.7 |
| 21 | -16.2 | 10.9 |
| 22 | -15.5 | 9.4 |
| 23 | -14.6 | 9.5 |
| 24 | -14.5 | 10.9 |
| 25 | -13.9 | 9.3 |
| 26 | -15.1 | 10.3 |
| 27 | -14.0 | 9.1 |
| 28 | -14.1 | 10.1 |
| Gemsbok (*Oryx gazella*) | | |
| 1 | -13.9 | 8.1 |
| 2 | -14.9 | 8.3 |
| 3 | -13.7 | 7.6 |
| 4 | -15.1 | 7.6 |
| 5 | -14.6 | 8.4 |
| 6 | -13.3 | 9.3 |
| 7 | -13.1 | 9.9 |
| 8 | -13.2 | 11.7 |
| 9 | -15.7 | 15.6 |
| 10 | -13.6 | 14.1 |
| 11 | -13.3 | 13.0 |
| 12 | -14.0 | 13.6 |
| 13 | -14.5 | 10.0 |
| 14 | -14.4 | 9.5 |
| 15 | -11.9 | 9.2 |
| 17 | -14.0 | 9.2 |
| 18 | -15.2 | 9.6 |
| 19 | -15.0 | 9.4 |
| 20 | -14.6 | 10.0 |
| 21 | -13.9 | 9.4 |
| 22 | -13.9 | 9.0 |
| 23 | -14.8 | 9.5 |
| 24 | -14.9 | 9.6 |
| 25 | -13.2 | 9.5 |
| 26 | -13.5 | 9.4 |
| 27 | -14.3 | 9.2 |
| 28 | -13.7 | 10.4 |
| 29 | -14.2 | 9.1 |
| 30 | -13.8 | 8.8 |
| 31 | -13.5 | 15.4 |
| Guinea fowl (Numida meleagris) | | |
| 1 | -18.5 | 9.0 |
| 2 | -15.4 | 8.5 |
| 3 | -15.0 | 10.9 |
| 4 | -14.9 | 9.3 |
| Hartebeest (*Alcelaphus buselaphus*) | | |
| 1 | -13.2 | 6.8 |
| 2 | -13.5 | 8.7 |
| 3 | -13.3 | 6.9 |
| 4 | -14.3 | 8.0 |
| 5 | -11.8 | 7.3 |
| 6 | -13.8 | 8.5 |
| 7 | -11.3 | 10.1 |
| 9 | -13.1 | 9.9 |
| 10 | -10.4 | 10.6 |
| 11 | -12.2 | 8.1 |
| 12 | -12.2 | 11.6 |
| 13 | -12.9 | 7.9 |
| 14 | -11.1 | 7.8 |
| 15 | -10.7 | 6.5 |
| 17 | -10.9 | 5.9 |
| Springhare (*Pedetes capensis*) | | |
| 1 | -11.6 | 12.7 |
| 2 | -15.7 | 9.6 |
| 3 | -11.6 | 10.4 |
| 3 | -14.8 | 10.3 |
| 4 | -13.6 | 8.7 |
| 5 | -14.2 | 9.4 |
| 6 | -11.7 | 11.9 |
| 7 | -11.8 | 12.6 |
| 8 | -14.3 | 11.9 |
| 9 | -12.0 | 9.5 |
| Warthog (*Phacochoerus africanus)* | | |
| 1 | -11.8 | 8.5 |
| 2 | -11.7 | 7.4 |
| 3 | -16.2 | 6.4 |
| 4 | -10.9 | 6.4 |
| 5 | -12.1 | 6.7 |
| 6 | -12.1 | 6.7 |
| 7 | -11.0 | 9.0 |
| 8 | -11.9 | 6.3 |
| 9 | -12.5 | 9.7 |
| 10 | -13.6 | 9.1 |
| 11 | -11.4 | 6.8 |
| 12 | -12.5 | 8.1 |
| 13 | -11.7 | 7.0 |
| 14 | -12.6 | 8.5 |
| Kudu (*Tragelaphus strepsiceros*) | | |
| 1 | -22.8 | 6.4 |
| 2 | -22.5 | 10.2 |
| 3 | -21.9 | 7.6 |
| 4 | -21.4 | 6.9 |
| 5 | -22.8 | 11.2 |
| 6 | -20.9 | 7.0 |
| 7 | -22.3 | 13.4 |
| 8 | -22.0 | 10.3 |
| 9 | -22.2 | 11.1 |
| 10 | -22.1 | 10.4 |
| Scrub hare (*Lepus saxatilis*) | | |
| 1 | -17.1 | 6.7 |
| 2 | -20.5 | 8.1 |
| 3 | -20.5 | 9.9 |
| Springbok (*Antidorcas marsupialis*) | | |
| 1 | -26.0 | 8.6 |
| 2 | -22.0 | 9.1 |
| 3 | -21.4 | 10.4 |
| 4 | -21.5 | 10.0 |
| 5 | -21.2 | 9.8 |
| 7 | -20.7 | 10.1 |
| 8 | -20.8 | 10.6 |
| Eland (*Taurotragus oryx*) | | |
| 1 | -22.7 | 9.6 |
| 2 | -22.7 | 9.0 |
| Steenbok (*Raphicerus campestris*) | | |
| 1 | -23.0 | 7.2 |
| 2 | -24.6 | 8.5 |
