# Supplementary material for: The Conflict between Cheetahs and Humans on Namibian Farmland Elucidated by Stable Isotope Diet Analysis
Source: PLoS One. 2014 Aug 27;9(8):e101917. doi: 10.1371/journal.pone.0101917 (PMC4146470; doi:10.1371/journal.pone.0101917)
Supplement: Table S2 — Raw isotopic data of cheetahs (1 = solitary males, 2 = males of bachelor groups, 3 = females. (DOC) [file pone.0101917.s002.doc]

Table S2:

| **Cheetah group** | **δ^13^C (‰)** | **δ^15^N (‰)** |
| --- | --- | --- |
| 1 | -20.2 | 10.0 |
| 1 | -13.1 | 11.1 |
| 1 | -12.7 | 11.3 |
| 1 | -17.8 | 11.2 |
| 1 | -13.1 | 11.0 |
| 1 | -19.2 | 12.2 |
| 1 | -19.0 | 9.8 |
| 1 | -10.7 | 10.0 |
| 1 | -10.3 | 11.1 |
| 1 | -16.9 | 10.7 |
| 1 | -20.7 | 11.3 |
| 1 | -17.7 | 10.5 |
| 1 | -15.6 | 10.5 |
| 1 | -19.8 | 12.1 |
| 1 | -18.1 | 13.3 |
| 1 | -17.2 | 10.2 |
| 1 | -16.1 | 11.5 |
| 1 | -18.4 | 11.1 |
| 1 | -15.9 | 10.6 |
| 1 | -14.2 | 9.7 |
| 1 | -13.7 | 9.5 |
| 2 | -11.2 | 10.2 |
| 2 | -11.5 | 10.6 |
| 2 | -19.2 | 11.1 |
| 2 | -14.8 | 11.7 |
| 2 | -10.7 | 9.9 |
| 2 | -10.5 | 10.9 |
| 2 | -11.0 | 11.9 |
| 2 | -13.7 | 10.4 |
| 2 | -15.2 | 11.4 |
| 2 | -12.0 | 11.0 |
| 2 | -16.3 | 13.1 |
| 3 | -20.0 | 11.0 |
| 3 | -18.8 | 10.1 |
| 3 | -19.8 | 11.4 |
| 3 | -17.7 | 10.2 |
| 3 | -17.1 | 10.6 |
| 3 | -19.0 | 11.7 |
| 3 | -13.3 | 12.6 |
| 3 | -17.6 | 12.3 |
| 3 | -18.0 | 11.0 |
