# Supplementary material for: The Conflict between Cheetahs and Humans on Namibian Farmland Elucidated by Stable Isotope Diet Analysis
Source: PLoS One. 2014 Aug 27;9(8):e101917. doi: 10.1371/journal.pone.0101917 (PMC4146470; doi:10.1371/journal.pone.0101917)
Supplement: Table S3 — Raw isotopic data of 4 tissues in 9 cheetahs (1 = female, 8 males), rbc = red blood cells. (DOC) [file pone.0101917.s003.doc]

Table S3:

| **Ind.** | **δ^13^C (‰)** | | | | **δ^15^N (‰)** | | | |
| --- | --- | --- | --- | --- | --- | --- | --- | --- |
|  | **fur** | **muscle** | **rbc** | **plasma** | **fur** | **muscle** | **rbc** | **plasma** |
| 1 | -12.7 | -14.8 | -13.7 | -14.5 | 11.4 | 10.7 | 10.4 | 11.2 |
| 2 | -15.2 | -15.9 | -15.2 | -13.3 | 13.2 | 12.8 | 11.4 | 12.4 |
| 3 | -15.6 | -16.9 | -16.1 | -18.9 | 13.0 | 12.8 | 11.5 | 13.7 |
| 4 | -16.7 | -18.7 | -18.4 | -18.7 | 12.0 | 11.2 | 11.1 | 12.4 |
| 5 | -18.1 | -16.4 | -18.0 | -14.7 | 14.5 | 11.8 | 11.0 | 12.8 |
| 6 | -13.8 | -14.5 | -14.2 | -11.5 | 11.9 | 10.3 | 9.7 | 12.0 |
| 7 | -12.2 | -14.6 | -13.7 | -15.7 | 10.7 | 10.1 | 9.5 | 11.3 |
| 8 | -10.3 | -12.0 | -12.0 | -12.0 | 12.2 | 11.1 | 11.0 | 12.6 |
| 9 | -18.6 | -17.7 | -16.3 | -18.5 | 13.0 | 12.2 | 13.1 | 11.7 |
